# Amyotrophic Lateral Sclerosis Is Associated with Hypolipidemia at the Presymptomatic Stage in Mice

**DOI:** 10.1371/journal.pone.0017985

**Published:** 2011-03-25

**Authors:** Sung-Min Kim, Heejaung Kim, Jee-Eun Kim, Kyung Seok Park, Jung-Joon Sung, Seung Hyun Kim, Kwang-Woo Lee

**Affiliations:** 1 Department of Neurology, College of Medicine, Seoul National University, Seoul, Korea; 2 Department of Neurology, College of Medicine, Hanyang University, Seoul, Korea; Okayama University Graduate School of Medicine, Dentistry and Pharmaceutical Sciences, Japan

## Abstract

**Objective:**

To demonstrate that hypolipidemia is a typical feature of the mouse model of amyotrophic lateral sclerosis (ALS) and to assess the association between hypolipidemia and disease stage, dietary intake, and sex.

**Methods:**

We compared daily dietary intake, body weight, and serumlipid and glucose levels in ALS mice and wild-type controls at different stages of the disease.

**Findings:**

Total cholesterol low-density lipoprotein (LDL) and LDL/high-density lipoprotein (HDL) ratio were significantly lower in ALS mice compared with controls. Subgroup analysis revealed that the incidence of hypolipidemia was significantly greater in male, but not female, ALS mice compared with control mice and that hypolipidemia was present at the presymptomatic stage of the disease. This hypolipidemia can be found without a decrease in the serum levels of other energy sources, such as glucose, in the presymptomatic stage.

**Conclusions:**

Hypolipidemia is present at the presymptomatic stage of the ALS mouse model in the absence of malnutrition, significant neuromuscular degeneration or regeneration, and respiratory difficulty. Our findings suggest that hypolipidemia might be associated with the pathomechanism of ALS and/or lipid-specific metabolism rather than simply an epiphenomenon of neuromuscular degeneration or energy imbalance.

## Introduction

Amyotrophic lateral sclerosis (ALS) is an adult-onset progressive neurodegenerative disease that affects the motor neurons of the cerebral cortex, brainstem, and spinal cord and typically causes death within 5 years from onset [1], [2]. It has long been recognized as a disease of the motor neurons; however, increasing evidence suggests the involvement of extra-motor neurons [3], [4] and extraneuronal tissue in the pathogenesis of ALS [1], [5]. Recent studies have detected abnormal lipid metabolism in ALS and investigated its cause [6]–[8], its role in the disease progression [9], [10], and its association with other systemic factors [10].

Despite the finding of abnormal lipid metabolism in ALS, conflicting results have been reported for basal serum lipid levels [8], [9], [10], the cause of dyslipidemia [7], [10], and the relationship between serum lipid levels and respiratory function and/or disease progression [10]. Moreover, it is unclear whether the dyslipidemia is the result of a specific need for lipids or a non-specific need for energy, and the role of serum lipid in ALS is further confused because several studies do not distinguish between these hypotheses [8], [11], [12].

We hypothesized that hypolipidemia in ALS is related to the pathogenesis of the disease and not secondary to peripheral neuromuscular system or respiratory function. Furthermore, we hypothesized that the hypolipidemia is independent of the depletion of other energy sources such as glucose.

## Methods

### ALS mice

B6SJL-Tg(SOD1-G93A)1Gur/J mice with a high copy number of transgenic human mutant superoxide dismutase 1s (SOD1s), which contained a glycine 93 (Gly93) to alanine (Ala) substitution (Strain No. 002726), were obtained from the Jackson Laboratory (Bar Harbor, ME, USA) [13]. The mice were housed under a 12-h light/dark cycle and bred as per the supplier's protocol [14]. The presence of the human *G93A* transgene was confirmed with polymerase chain reaction (PCR) assays using DNA extracted from the tail tissue. In this strain, the first ALS symptoms and end-stage symptoms appear at approximately 77 days and 136 days of age, respectively [15], [16]; thus, we divided 30 transgenic mice (15 male) into three equal-sized age groups: 60 (presymptomatic), 90 (early symptomatic), and 120 days (late symptomatic). They were fed a chow diet (LAB rodent CHOW; 38057; Cargill Agri Purina, Inc, Korea) Thirty age- and sex-matched wild-type mice served as controls. The mice in each age group were fasted for 6 h [17] and euthanized using deep inhalation of isoflurane, and then blood was collected via cardiac puncture. The blood was centrifuged and the serum was preserved in a deep freezer. Serum cholesterol (Catalog NO: HC0721/0722), low-density lipoprotein (LDL, HC2210), high-density lipoprotein (HDL, HC2110), and triglycerides (TG, HC0821/0822), were quantified using enzymatic assays (HBI Co., Anyang, Korea). Protein (HC1421) and glucose (HC0621/0622) levels were quantified using the Biuret assay and Trinder glucose activity test, respectively. The daily dietary intake of each mouse was measured for 5 consecutive days before the mouse was euthanized, and body weight was measured at 1 and 5 days before the mice were euthanized.

The animal study procedures were approved by the Institutional Animal Care and Use Committee (IACUC) of Hanyang University. (Permit number: 09-032).

### Statistical analyses

We compared the mean serum levels of cholesterol, TG, LDL, HDL, and glucose in the ALS and control mice using the Mann–Whitney *U*-test. Statistical analyses were performed using the Statistical Package for Social Sciences (SPSS) version 17.0, and *p*<0.05 was considered to be statistically significant. Results are expressed as mean ± standard deviation, throughout the text.

## Results

### Hypolipidemia in the ALS mice of all age and age-sex matched controls. (Figure 1, left panel)

**Figure 1:**
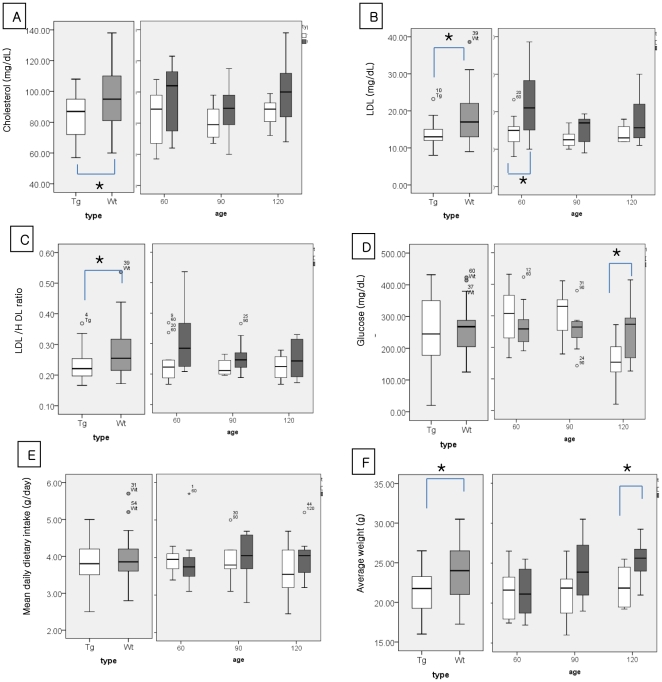
Basal serum levels of cholesterol (A), low-density lipoprotein (B), LDL/HDL ratio (C), glucose (D), and mean daily dietary intake (E), and average weight (F). The left panels show the combined value for ALS mice of all age (Tg, n = 30) and the control mice (Wt). The right panels show the values for the ALS mice and controls in each age group. (n = 10, for each group) Values in the boxes and whisker plots are median values and interquatile ranges respectively. LDL: low-density lipoprotein, HDL: high-density lipoprotein. * *p*<0.05.

A comparison of the all pre-symptomatic, early symptomatic, and late symptomatic ALS mice (n = 30) and control mice (n = 30) showed that the ALS mice had significantly lower serum cholesterol (83.45 mg/dL±12.83 vs. 94.29±19.75 mg/dL, respectively; *p* = 0.011), LDL (13.57 mg/dL±3.13 vs. 17.90±7.23 mg/dL, *p* = 0.003), and LDL/HDL ratio (0.22±0.05 vs. 0.26±0.08, *p* = 0.010) than did the control mice (Figure. 1, left panel). Dietary intake did not differ between the ALS mice and controls (3.79±0.51 g/day vs. 3.93±0.62 g/day, *p = 0.543*), suggesting that the hypolipidemia was not the result of dysphagia or poor dietary intake (Figure. 1,A–C,E, left panel).

### Hypolipidemia in the pre-symptomatic ALS mice and age-sex matched controls. (Figure 1, right panel)

The analysis of serum levels of lipid, glucose, dietary intake and body weight were also performed according the different disease stage in ALS mice and controls. (Figure 1, right panel) In the presymptomatic stage (60 days after birth; n = 10), the serum LDL level was significantly lower in the ALS mice than in the age- and sex-matched controls (14.50±4.45 mg/dL vs. 21.68±9.45 mg/dL, respectively; *p* = 0.043). Furthermore, the serum cholesterol and the LDL/HDL ratio were lower in the presymptomatic group than in the controls; however, the values did not reach statistical significance. Dietary intake in the presymptomatic ALS group did not differ from that of the age- and sex-matched controls. (3.90±0.30 g/day vs. 3.86±3.86 g/day, *p = 0.342*)

### Hypolipidemia and non-specific energy demands

We found no significant difference in serum glucose levels between the ALS mice of all age (n = 30) and control groups (252.09±109.70 mg/dL vs. 263.83±74.50 mg/dL, *p* = *ns*), suggesting that the hypolipidemia was not attributable to non-specific energy demands. (Figure 1D, left panel) The ALS mice were hypoglycemic, compared with the controls, only in the late symptomatic stage, (153.00±72.34 mg/dL vs. 250.50±85.66 mg/dL, *p* = 0.015) when they may have experienced excessive metabolic demands for muscle degeneration/regeneration and respiratory compensation. [10], [18], (Figure. 1D, right panel).

### Hypolipidemia and sex

**Figure 2:**
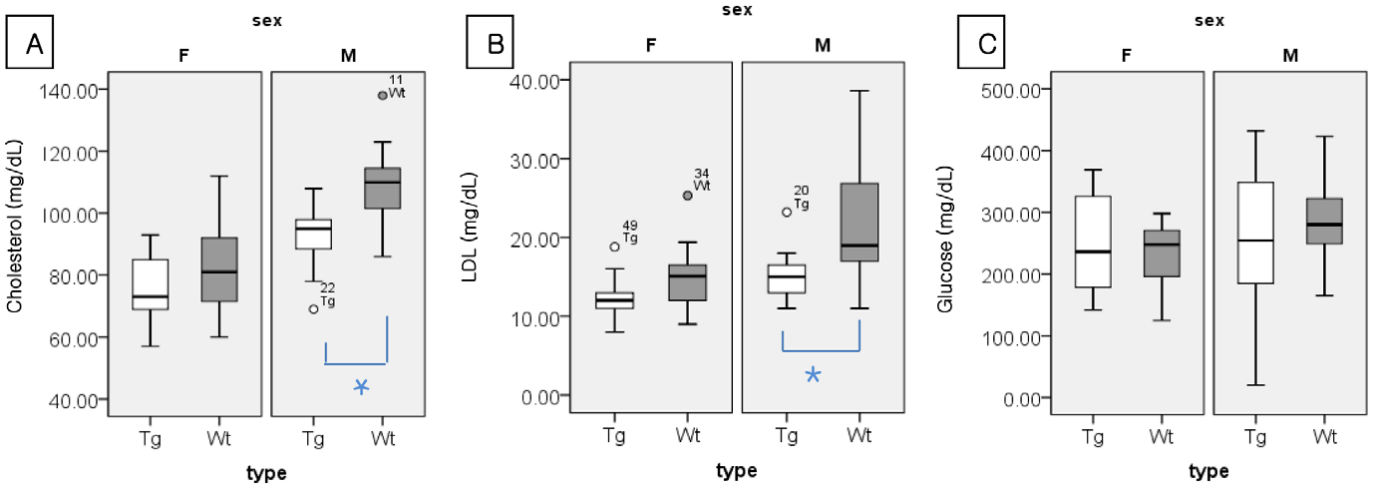
Basal serum levels of cholesterol (A), low-density lipoprotein (B), and glucose (C) according to sex in ALS mice of all age (Tg) groups (n = 15, for each group) and age- and sex-matched controls (Wt). The basal serum cholesterol and low-density lipoprotein were significantly lower in male ALS mice compared with control mice. Values in the boxes and whisker plots are median values and interquatile ranges respectively. LDL: low-density lipoprotein, HDL: high-density lipoprotein. * *p*<0.05.

Basal serum cholesterol (92.00±9.55 mg/dL vs 108.60±13.24 mg/dL, *p* = 0.0 1) and LDL (15.08±2.98 mg/dL vs. 21.63±7.94 mg/dL, *p* = 0.012) were significantly lower in all (including pre-symptomatic, early symptomatic, and late symptomatic) male ALS mice (n = 15) compared with the sex-matched controls. (figure 2; right panel), but not in female ALS mice. (figure 2; left panel) Hypolipidemia in the male ALS mice developed in the absence of significant hypoglycemia (Figure. 2C, right panel).

**Figure 3:**
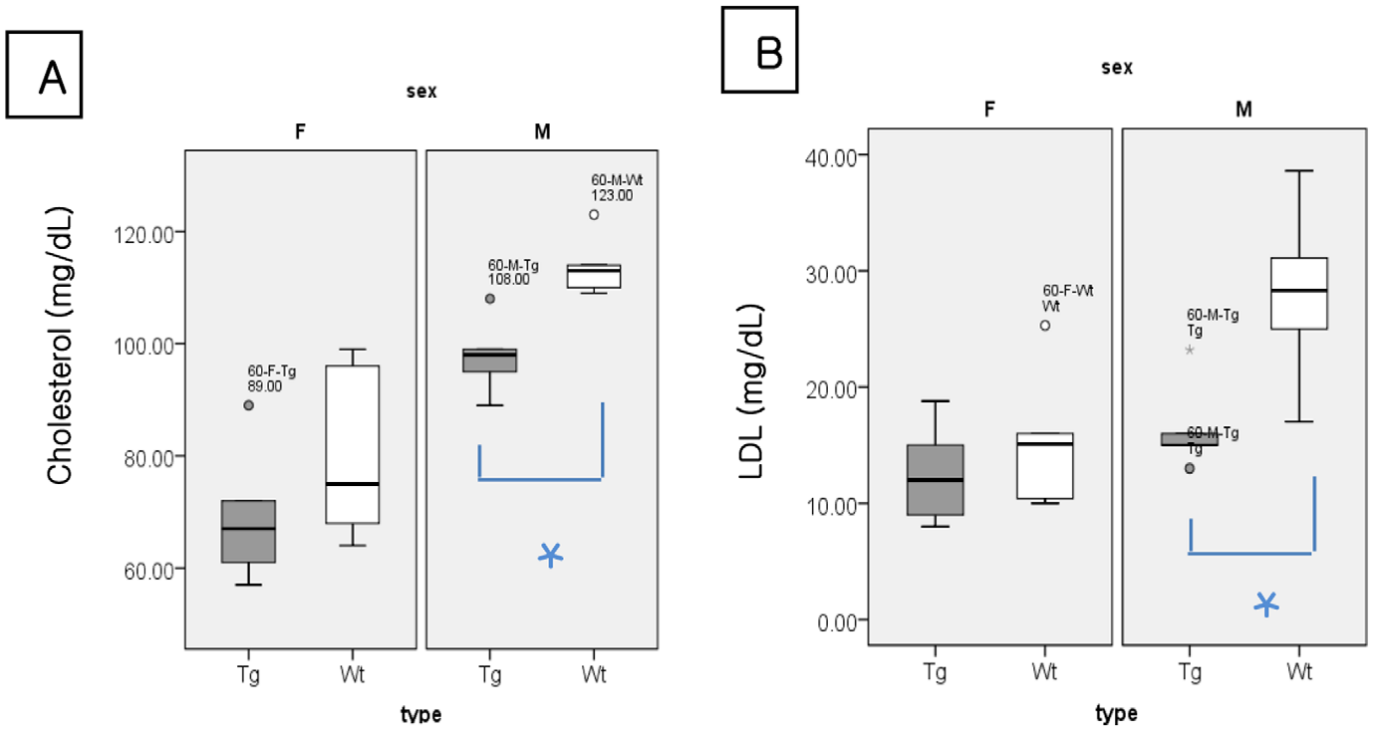
The basal serum levels of cholesterol (A) and low-density lipoprotein (B) in female (left panel) and male (right panel) pre-symptomatic ALS (Tg, aged 60 days) and control (Wt) mice. (n = 5, for each group) The basal serum cholesterol and low-density lipoprotein levels were significantly lower in male (right panel), but not in female ALS mice (left ) compared with control mice. Values in the boxes and whisker plots are median values and interquatile ranges respectively. LDL: low-density lipoprotein. * *p*<0.05.

The comparison of presymptomatic male mice (age 60 days, *n* = 5) and their age- and sex-matched controls (Figure 3) showed that the ALS mice had significantly lower serum cholesterol (97.80±6.91 mg/dL vs. 113.80±5.54 mg/dL, *p* = 0.008) and LDL (16.44±3.93 mg/dL vs. 28±7.93 mg/dL, *p* = 0.016). Though presymptomatic female mice group (Figure 3, left panel) and early and late symptomatic mice groups of both sexes (data not shown) showed a tendency toward lower serum cholesterol and LDL statistical significance was not reached.

## Discussion

In the present study, we observed that (1) hypolipidemia occurred in ALS mice; (2) the hypolipidemia was not associated with a decrease in the level of serum glucose, another important source of energy; (3) significant hypolipidemia was observed in male, but not female mice; and (4) hypolipidemia was present at the presymptomatic stage of ALS, particularly in males, when no significant neuromuscular degeneration or respiratory insufficiency was present.

Our findings are consistent with (1) studies that have reported an increase in lipid clearance [7], suggesting that a high-fat diet might be beneficial [8], (2) reports of high metabolic demands in the early disease stage [6], [18], [19], suggesting that interference with lipid metabolism can cause ALS-like symptoms [20], and (3) reports of a sex effect in the progression [21] or onset of symptoms [20] in ALS mice. However, our findings are not consistent with those of two recent retrospective studies: Dupuis et al. [9] observed hyperlipidemia in patients with ALS, and Chiò et al. [10] suggested that hypolipidemia in patients with ALS was associated with respiratory insufficiency. The inconsistency between our findings and these reports may merely reflect a difference between the SOD1 mutant ALS mice model and patients with ALS; [22] however, other factors may account for these discrepancies. First, the dietary intake of patients with ALS may be affected by several factors including dysphagia, severe cognitive dysfunction (e.g., frontotemporal lobar dementia), depressive mood, and the presence of a percutaneous endoscopic gastrostomy (PEG) or a Levin tube for feeding which variables were not controlled for analysis in previous study on patients with ALS [9]. Second, hypermetabolism, which can affect serum lipid levels, has been reported to be more severe in male than in female patients [6]; thus, studies of serum metabolites such as lipid and glucose should be analyzed according to sex. However, Dupuis et al. [9] and Chiò et al. [10] did not assess sex differences in lipid and glucose levels. Finally, fasting time has a significant influence on basal serum lipid levels, and the presence or absence of fasting can affect the relative level of serum lipid in patients with ALS compared with control patients [8]. Dupuis et al. did not clearly describe the patient fasting time.

The results of our study indicate that hypolipidemia in ALS mice is not associated with decreased food intake, malnutrition, demand for non-specific energy generation [11], degeneration or regeneration of muscle [8], or respiratory insufficiency [10] for the following reasons: hypolipidemia was observed in ALS mice that had normal food intake; the basal serum glucose level, an important energy source that may be affected by the nutritional intake, did not differ between ALS and control mice despite the presence of hypolipidemia in the ALS mice; and hypolipidemia was observed at the presymptomatic stage, particularly in male mice (Figure 1B and 3), when neither significant motor weakness nor muscle degeneration was present [15].

The serum glucose levels in the ALS mice were not significantly different from those of the controls until the late symptomatic stage (Figure. 1D, right panel). At this stage, the decrease in serum glucose may have been the result of an increased need for regeneration of the neuromuscular system or a high demand to compensate for respiratory muscle weakness. However, this finding does not alter our conclusion because the ALS mice were euglycemic as well as hypolipidemic at the presymptomatic stage.

In the present study, the male, but not the female, ALS mice showed statistically significant hypolipidemia compared with the age-and sex-matched controls (Figure 2). The reasons for this sex difference are not clear and may reflect lack of statistical power as numbers in each group (n = 5) were small. However, previous studies have shown that knockout of the liver X receptor β (*LXRβ*) gene, which is associated with cholesterol metabolism in the central nervous system (CNS), produced ALS symptoms and resulted in motor neuron degeneration in male, but not female, mice [21]. Furthermore, hypermetabolism has been reported to be more severe in male, compared with female, patients with ALS [18], and estrogen has been shown to delay the disease progression in ALS mice [20]. These findings indicate possible differences in metabolism between men and women with ALS.

The factors underlying the increased demand for lipids and/or hypolipidemia in ALS are not known. Previous studies have suggested that the skeletal muscle activity is the cause of hypermetabolism in ALS; however, that hypothesis was based on results showing an increase in glucose uptake rather than lipid levels [8]. A lipid-specific metabolic abnormality in ALS is suggested by our results, together with studies showing that a high-fat diet may alter the CNS pathology (i.e., death of motor neurons) [8] and that inactivation of the *LXRβ* gene, which participates in cholesterol transport from glial cells to neurons in the CNS, caused motor neuron degeneration in mice [21], as well as by evidence that the CNS, as well as the skeletal muscles, may contribute to the high lipid demand which might be associated with ALS pathogenesis [7].

Our study has several limitations. First, the number of mice in the study was small; thus, although the ALS mice in the presymptomatic group showed a tendency toward lower serum cholesterol and LDL/HDL ratio, statistical significance was not reached. Second, we demonstrated that ALS mice are hypolipidemic, but could not identify the underlying mechanism. Last, our data might have been more valuablehad we been able to take sequential serum samples from the same mice as the disease progress. However, because the volume of serum in ALS mice were too small (especially in 60 days after birth), we could only get a large enough volume of serum taken at three different specific time points, by cardiac puncture after general anesthesia, which inevitably led to the sacrifice of mice due to circulatory collapse.

Nevertheless, our study confirmed the finding that ALS is associated hypolipidemia prior to the onset of symptoms and a decrease in respiratory function [8], [10] and showed a sex difference in the metabolic abnormalities of ALS mice. Furthermore, our findings suggest a lipid-specific metabolic deterioration in ALS because hypolipidemia is observed in the absence of hypoglycemia, particularly at the presymptomatic stage. Together these findings suggest that hypolipidemia in ALS is not an epiphenomenon of neuromuscular degeneration, respiratory difficulty, or energy imbalance, but might be associated with the disease pathomechanism and/or lipid-specific metabolism. Further studies are required to determine the cause, roles, and sex differences associated with the high lipid demand in ALS.
